# New clinical application of amniotic membrane transplant for ocular surface disease

**Published:** 2016

**Authors:** A Gheorghe, M Pop, M Burcea, M Serban

**Affiliations:** * “Carol Davila” University of Medicine and Pharmacy, Bucharest, Romania; ** Clinical Emergency Eye Hospital, Bucharest, Romania; *** ”Victor Anastasiu” National Institute for Aeronautical and Spatial Medicine, Bucharest, Romania

**Keywords:** ocular surface, amniotic membrane transplantation, limbal stem cells deficiency

## Abstract

The new defined anatomical and functional complex conjunctiva-limbus-cornea is a new concept, which helps clinicians better understand and treat ocular surface pathologies. The management of the ocular surface disease has changed dramatically over the years, with spectacular improvements of techniques, and of course, results. The amniotic membrane, used as a graft or as a substrate for the cultivation of limbal corneal cells has showed encouraging results.

**Aim:** To investigate the usefulness of amniotic membrane transplantation in ocular surface pathologies.

**Method:** The study is retrospective. 28 eyes of 28 patients with ocular surface pathologies (ocular burns, recent and old, symblepharon, pterygium, corneal and conjunctival tumors, persistent epithelial defect) underwent an amniotic membrane transplantation during a 1 year period. The necrotic and the scar tissue were first excised in all the patients and the amniotic membrane was sutured with an epithelial face up. Follow up ranged from 1 to 12 months.

**Results:** Good results were obtained in all 28 eyes. The anatomy of cornea and conjunctiva was improved, with limited benefits only in old ocular burn, symblepharon and in one case of extended tumors of the cornea and conjunctiva. Out of 28 eyes, 23 (82%) also had a visual acuity improvement.

**Conclusion:** The amniotic membrane may be considered a good alternative for ocular surface reconstruction especially in acute status.

**Abbreviations:** AMT = amniotic membrane transplantation, BCVA = best corrected visual acuity, BUT = break up time

## Introduction

The management of severe ocular surface disease has changed dramatically over the past 25 years. All the patients had a poor prognosis and the only available tools were artificial tears, penetrating keratoplasty, and tarsorrhaphy. Nowadays, new concepts and new treatments are emerging due to the better understanding of the ocular surface concept. Advances in microsurgical techniques and the understanding of the role of the limbal stem cells have led to great improvements in both visual acuity and the quality of life of these patients [**1**].

The surface of the eye is an extraordinary and vital component of vision. The smooth wet surface of the cornea is the major refractive surface of the visual system, which, along with corneal transparency, enables light to proceed through the lens and onto the retina for photoreceptor activation. The presence of the smooth wet, refractive ocular surface required for vision comes, however, at a cost [**2**].

The maintenance and protection of the smooth refractive surface of the cornea is the function of the Ocular Surface System.

The ocular (eye) surface includes two major territories, the cornea, and the conjunctiva, bordered by the upper and the lower lids. Unlike the skin covering the rest of the body, a thin layer of tear film covers the ocular surface. A stable tear film present when the eye is open is the key mechanism to maintain the ocular surface health [**2**].

Patients with ocular surface diseases suffer from loss of vision, discomfort, infection, erosion, ulceration, and destruction with a scarring of the eye surface.

Amniotic membrane transplantation has a long tradition in ophthalmic surgery and has recently become very popular because of newly developed methods of tissue preservation. Amniotic membrane can function as a basement membrane substitute or as a temporary graft in the eye. It has anti-inflammatory and anti-scarring effects and contains growth factors that promote epithelial wound healing on the surface of the eye [**3**]. Amniotic membrane transplantation has been found to be a good alternative for corneal and conjunctival reconstruction in many clinical situations, including acute burns [**4**], persistent epithelial defects of the cornea [**5**], and diseases that cause conjunctival scarring [**6**]. In fact, almost all these ocular surface pathologies lead to limbal stem cell deficiency [**7**,**8**]. A rapid therapeutic approach using amniotic membrane transplantation, for its properties or as a substrate for cultivating limbal epithelial corneal cells has proven benefic, showing good results. Ganger et al. have followed case records of patients with limbal stem cell deficiency for over 10 years, who underwent limbal epithelial transplantation on an amniotic membrane graft. The anatomical success and visual improvement was seen in almost all patients, adults, and children [**9**].

## Method

Our study was conducted on 28 eyes of 28 patients. 9 of them had alkali burns, 5 were recent and 4 were old. 2 patients had symblepharon, 6 pterygium with important corneal invasion. We also enrolled 8 patients with tumors of the cornea and conjunctiva, 2 of them presenting a large invasion of the surrounding tissue, a persistent epithelial defect in 1 young patient without any other systemic pathologies and diabetes mellitus in 2 patients. All the patients complained of photophobia, epiphora, decreased visual acuity, and poor cosmetic status. BCVA ranged from LogMAR 0 to LogMAR 1 in patients with severe corneal tumors, symblepharon and old alkali burns. All the patients were carefully examined at the ocular slit biomicroscope. Schirmer tests, BUT tests and corneal staining with fluorescein examination for corneal epithelial defects were performed. Before the surgery, all the patients filled a questionnaire ranging the score of their photophobia and general ocular discomfort. The amniotic membrane was carefully prepared and after the removal of necrotic or scaring tissue of the patient, it was sutured, on the eye, with epithelial face up. After surgery, the patients were examined at 1, 7, 14 days and then monthly. They were asked to fill in the questionnaire again 1 month after the surgery.

These individual data were analyzed and results were reported in percentage or absolute numerical value.

## Results

**Fig. 1:**
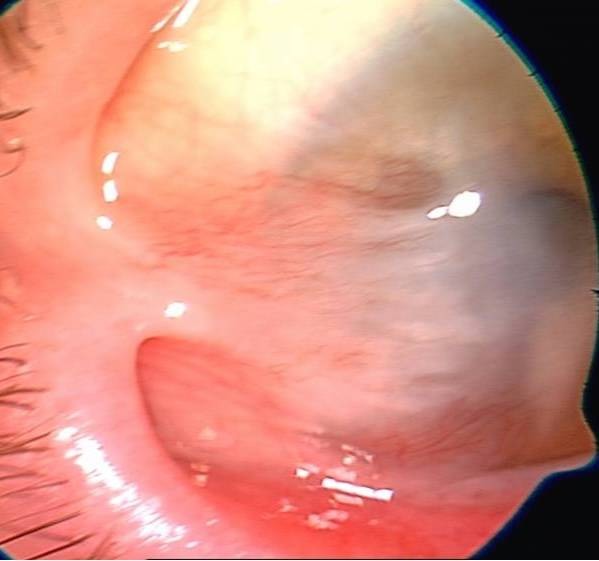
Symblepharon

**Fig. 2:**
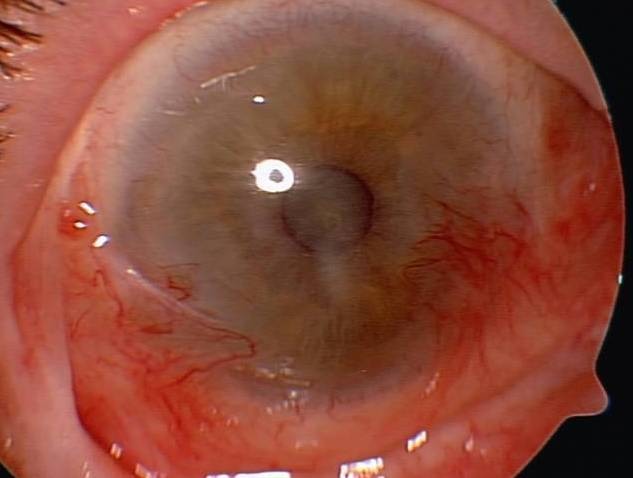
One year after AMT

**Fig. 3:**
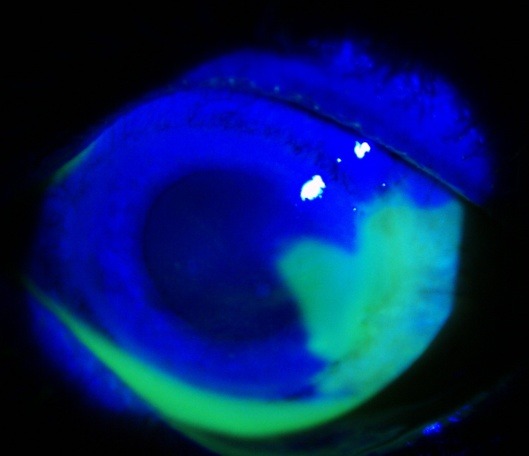
Acute corneo-limbal alkali burn

**Fig. 4:**
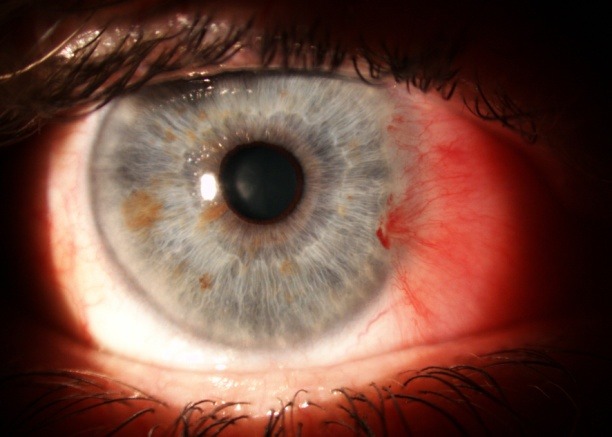
Four months after AMT

**Fig. 5:**
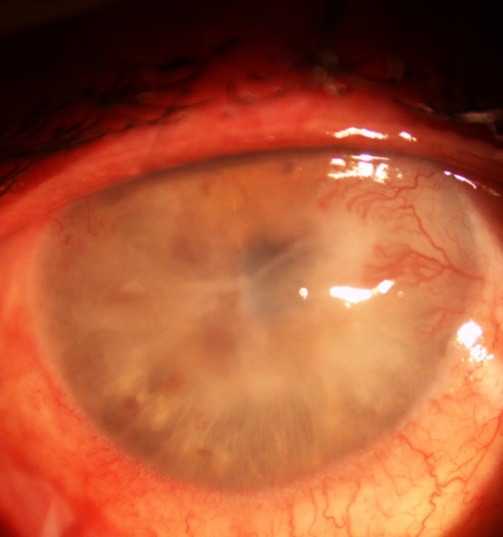
Old alkali burn with corneal neovascularization

**Fig. 6:**
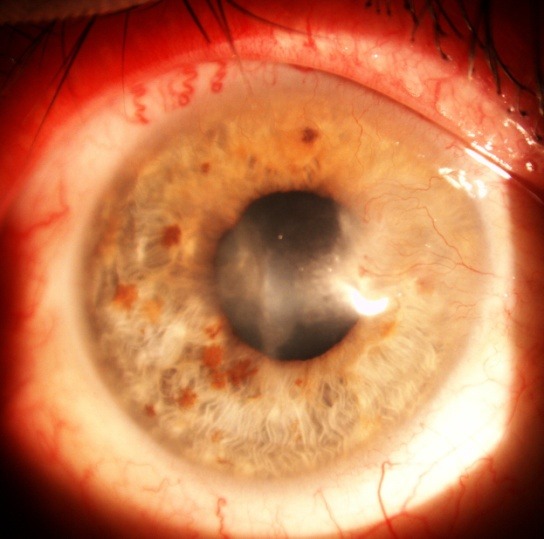
Two months after AMT

Good results were encountered in all 28 eyes. The anatomy of the cornea and conjunctiva was improved, with limited benefits only in old ocular burns, symblepharon (**Fig. 1**,**2**) and in one case of extended tumor of the cornea and conjunctiva. From 28 eyes, 23 (82%) also had a visual acuity improvement. The improvement was important in patients with a persistent epithelial defect and a recent/ acute alkali burn (**Fig. 3**,**4**), but the patients with old alkali burns and ocular tumors also had a benefit of 1 or 2 lines. The AMT BCVA ranged from LogMAR 0 to LogMAR 0.7. In our opinion, all the 28 patients had a good or improved cosmetic result, but only 26 were satisfied. Regarding the questionnaire, general discomfort symptoms improved in 25 patients, only one patient with symblepharon and 2 patients with tumors did not report an improvement. Photophobia was still present after AMT in 2 patients with old alkali burns (**Fig. 5**,**6**), 2 with symblepharon and 1 with ocular surface tumor, but the rest of 22 patients (78%) reported an improvement.

## Conclusions

The amniotic membrane transplantation is a good and viable option in the ocular surface diseases and reconstruction. Its properties and its function as a substrate for cultivating limbal epithelial corneal cells, opens a new era of regenerative medicine, giving hope to patients with poor prognosis. 
